# Taste alterations after hematopoietic cell transplantation: a scoping review

**DOI:** 10.1007/s00520-024-08900-w

**Published:** 2024-09-25

**Authors:** Caroline G. R. Dequae, Judith E. Raber-Durlacher, Joel B. Epstein, Ralph de Vries, Alexa M. G. A. Laheij

**Affiliations:** 1 Department of Oral Medicine, Academic Center for Dentistry Amsterdam, University of Amsterdam and VU University Amsterdam, Gustav Mahlerlaan 3004, 1081 HV Amsterdam, The Netherlands; 2 Department of Oral and Maxillofacial Surgery, Amsterdam UMC, University of Amsterdam, Amsterdam, The Netherlands; 3 Dental Oncology, Department of Surgery, City of Hope Comprehensive Cancer Center, Duarte, CA and Cedars-Sinai Medical System, Los Angeles, CA USA; 4 Medical Library, VU University, Amsterdam, The Netherlands

**Keywords:** Hematopoietic cell transplantation, Taste, Dysgeusia, Review

## Abstract

**Purpose:**

This review aimed to evaluate the prevalence and characteristics of dysgeusia after hematopoietic cell transplantation (HCT).

**Methods:**

A literature search (in PubMed, Embase.com and Web of Science) for clinical studies evaluating taste before and after HCT was performed up to June 22, 2023, in collaboration with a medical information specialist. After title and abstract review (N = 807) followed by full-text review (N = 61), articles that met the inclusion criteria were summarized in a table and synthesized narratively.

**Results:**

11 articles were analyzed in this review. All studies had a prospective design and patient populations included children (N = 3) and adults (N = 8) undergoing allogeneic or autologous HCT. Taste was assessed objectively (N = 6) and/or subjectively (N = 8) between baseline and 12 months after HCT. Before HCT, the self-reported (0–31%) and objective (2.4–10%) prevalence of dysgeusia was low. During the neutropenic phase, self-reported (20–100%) and objective (21.4%) dysgeusia was highest. In the post-engraftment period, the self-reported (18%) and objective (0–33%) prevalence of dysgeusia decreased. Different taste qualities were assessed in six studies including salt, sour, bitter, sweet, and umami.

**Conclusions:**

Some patients undergoing HCT experience dysgeusia prior to treatment. During the neutropenic phase, they had highest complaints, with recovery occurring in the post-engraftment period. All basic tastes, except bitter, were affected. Umami and salt were most affected during treatment. These findings have implications for patient management.

**Supplementary Information:**

The online version contains supplementary material available at 10.1007/s00520-024-08900-w.

## Introduction

Hematopoietic Cell Transplantation (HCT) is a widely used treatment, with The European Society for Blood and Marrow Transplantation (EBMT) reporting over 47, 412 performed transplants in Europe in 2021. Main indications, respectively for allogeneic and autologous HCT, include hematopoietic malignancies including leukemia, lymphoid and multiple myeloma. However, HCT can also be used for the treatment of solid tumors (e.g. neuroblastoma) or non-malignant disorders (e.g. multiple sclerosis) [1]. While offering a potentially curative outcome for a diverse range of conditions, survivors may develop short and long-term complications significantly impacting their quality of life (QoL) and life expectancy [2, 3]. Shortly after HCT, patients are at increased risk of opportunistic infections. Additionally, allogeneic HCT recipients are at risk of graft failure or developing Graft-versus-host disease (GvHD). Finally, throughout the entire post-transplant period, survivors remain at increased long-term risk for several diseases (e.g. cardiovascular disease; metabolic disorders) as well as relapse of the underlying disease, remaining a major cause of mortality [2].

Besides the previously mentioned challenges, patients undergoing HCT may also develop oral complications. The overall prevalence of oral complications in transplant recipients is estimated to be 80% [4, 5]. These complications can be either tissue specific (e.g. oral mucositis, salivary gland hypofunction, dysgeusia and caries) or non-tissue specific (e.g. increased bleeding and infection risk) [5].

Taste alterations, also known as dysgeusia, is an underappreciated and often overlooked complication of cancer treatment [6–8]. The sense of taste, in combination with smell, temperature and texture, play an important role in determining the overall flavor of food. Dysgeusia can affect the perception of all five basic tastes (sweet, sour, salt, bitter and umami) [6, 8, 9].

Different mechanisms, local and systemic, may be implicated in the etiology of taste alterations during cancer treatment. Chemotherapy and radiotherapy may cause direct taste and smell alterations by damaging taste receptor cells. Additionally, antineoplastic drugs may damage neuronal cells, modifying afferent taste pathways. [9–11]. In HCT recipients, taste may be adversely affected by other oral complications such as GvHD, oral mucositis, hyposalivation and/or oral infection [8]. In addition, several medications may contribute to altered taste [12]. There appears to be a pattern in the manifestation of oral complications after HCT, and evidence suggests that some of these problems may be interrelated [13]. However, the exact nature of these interactions causing taste abnormalities in HCT recipients remains unclear.

Dysgeusia is notably present during the active phase of treatment and may persist for days to months afterward [6, 14]. These changes can be evaluated both objectively and subjectively. Currently, there is no gold standard for the assessment of taste during cancer therapy [15]. Combining objective measures of taste function with well-validated patient-based outcome scales may provide valuable insight into the progression and characteristics of taste alterations after HCT [6, 7, 15], that may support future management of taste change. Patients experiencing taste alterations may derive less pleasure from eating and drinking and might avoid certain types of food. This can negatively affect their emotional state and nutritional intake resulting in a reduced QoL, malnutrition, dehydration, weight loss, fatigue and depressed mood. Ultimately, dysgeusia following HCT can negatively impact the overall recovery process [9, 13].

With an increasing number of survivors, there is a corresponding rise in patients with complications [3]. This emphasizes the growing importance of supportive care and complication management. Despite dysgeusia being an invalidating problem, its prevalence and characteristics after HCT remain unclear [6, 9]. Having a better understanding of dysgeusia after HCT is vital for effective patient management. Therefore, the aim of this scoping review is to comprehensively synthesize current available literature evaluating the prevalence and characteristics of dysgeusia after HCT in children and adults.

## Methods

This review is reported according to the PRISMA extension for Scoping Reviews (PRISMA-ScR) [16].

### Eligibility criteria

This scoping review included clinical studies with adult and pediatric patients undergoing allogeneic and/or autologous HCT. Studies reporting quantitative, qualitative and mixed-method taste characteristics were included. Furthermore, studies reporting baseline taste (i.e. before HCT) were included to correct for dysgeusia that is not related to HCT. Studies were excluded that investigated taste alterations not due to (the conditioning regimen of) HCT, animal and in vitro studies. Studies that did not provide baseline taste alterations were also excluded. Finally, certain publication types (editorials, reviews and case reports) were excluded.

### Search strategy

To identify the relevant publications, we conducted systematic searches in the bibliographic databases PubMed, Embase.com and Web of Science (Core collection) from inception to June 22, 2023, in collaboration with a medical information specialist. The following terms were used (including synonyms and closely related words) as index terms or free-text words: "Stem cell transplantation", "HSC", "HSCT", "Taste disorders".

The references of the identified articles were searched for relevant publications. Duplicate articles were excluded by a medical information specialist using Endnote X20.0.1 (Clarivate^tm^), following the Amsterdam Efficient Deduplication (AED)-method [17] and the Bramer-method [18].

The full search strategies for all databases can be found in the supplementary material.

### Selection process

Two reviewers (AL and CD) independently screened all potentially relevant titles and abstracts for eligibility using the review manager Rayyan QCRI [19]. If necessary, the full text article was checked for the eligibility criteria. Differences in judgement were resolved through a consensus procedure. Studies were included if they met the following criteria: (i) patients receiving HCT (allogeneic and/or autologous); (ii) studies reporting taste; (iii) clinical studies with adults and children; (iv) taste was measured at 2 different time points, including a baseline (i.e. before HCT) taste assessment. We excluded studies if they were: (i) studies investigating taste alterations not due to HCT or the conditioning regimen (e.g. Covid-19 or cytomegalovirus prophylaxis); (ii) animal and in vitro studies; (iii) certain publication types: editorials, reviews, case reports. Then one reviewer (CD) independently reviewed the full text of the remaining articles for final inclusion.

### Data extraction

Data were extracted from papers included in the scoping review by one reviewer (CD). The data extracted from selected articles included the number of participants, the type of conditioning regimen and transplant; and sample size to describe the general characteristics of the selected studies. To describe the prevalence and characteristics of taste, the reported presence of taste disorders, key taste outcomes, taste assessment time points and method of taste assessment were extracted.

### Data analysis and presentation

The selection of articles was visually presented in a flowchart. Data extracted from the included articles were synthesized in a structured table. Other relevant data were presented descriptively.

## Results

### Search results

**Fig. 1:**
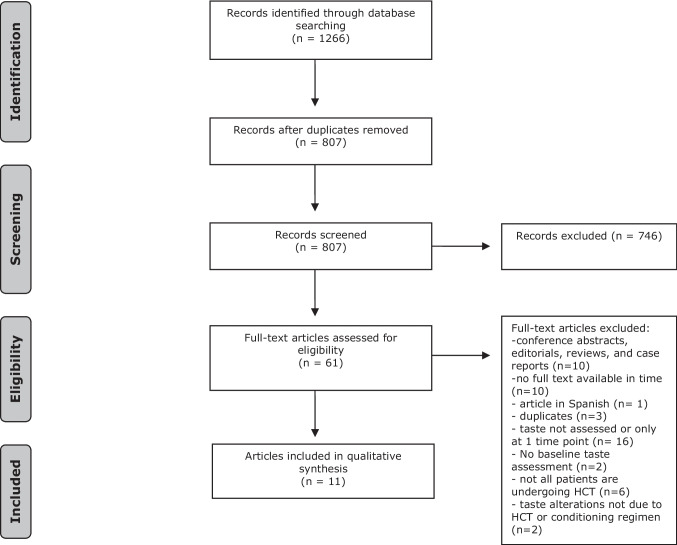
Flow diagram of the study selection process. Abbrevations HCT = hematopoietic cell transplantation

The literature search was performed up to June 22, 2023, and generated a total of 1266 articles: 263 in PubMed, 625 in Embase.com and 378 in Web of Science. After removing duplicates, 807 references remained. A total of 61 articles were retained for full-text review after screening the titles and abstracts of these publications according to the in- and exclusion criteria. The full text review led to the exclusion of 50 articles, of which the reasons are listed in Fig. 1. Finally, 11 articles were included in this review. This final selection was determined through consensus among CD and AL. The flow chart of the search and selection process is presented in Fig. 1.

### Characteristics of selected studies

From the 11 articles [20–30] (Table 1), a total of 494 patients were included with sample sizes ranging from 10 to 80 participants per study. Eight studies included adults [20, 21, 24, 25, 27–30] and three studies were performed in children [22, 23, 26]. All studies were designed prospectively, with one case-consecutive study [26].

**Table 1:** Summary of included articles

Author, year	Conditioning regimen	Type of transplant	Sample size	Overall presence of dysgeusia	Patients	Taste Outcome	Assessment method
Abaseed et al. 2018 [20]	Different	Allo + Auto	29	- BL (pre-HCT): 1.4 - Day 30: 30.4 - Day 80: 10.4 (self-report) *	Adults	- Decreased taste sensitivity for NaCl, citric acid on day 30 - Increased taste sensitivity for sucrose on day 30 - Taste sensitivity largely recovered on day 80	- CG (3 concentrations) - Self-report (EORTC QLQ-C30)
Andersson et al. 2009 [21]	Different (divided in MAC or RIC)	Allo	57	MAC: - BL (hospital admission): 9.0 - 1 month: 58.7 - 12 months: 17.7 RIC: - BL (hospital admission): 7.6 - 1 month: 32.0 - 12 months 14.4 (self report) *	Adults	- Change of taste in both groups, worst 1 month after SCT - MAC group showed more deterioration in change of taste at all time-points - Problems decreased over time in both groups	- Self-report (HDC-19)
Barale et al. 1982 [22]	TBI + methotrexate or cyclophosphamide + BMT	Not specified	11	- BL (hospital admission): 0% (n = 11) - Day 2: 100% (n = 11) - Day 45: 18% (n = 2) (self report)	Children (6-15y)	- Significant difference in sour threshold between patients and controls at admission - Minor changes in taste thresholds	- CG (9–11 concentrations) - Self-report (assessed verbally)
Cohen et al. 2012 [23]	Different	Allo + Auto	10	- BL (hospital admission): 10% - 1 month: 33% - 2 months: 0%	Children (8-15y)	- Taste dysfunction early in transplant was found to be transient - No taste dysfunction 2 months after HCT	- CG (5 concentrations)
Ferreira et al. 2020 [24]	Different	Allo + Auto	51	- BL(hospital admission): 2,4% - NP: 21,4%	Adults	- Significant increase in hypogeusia (most) and dysgeusia between BL and NP - Bitter taste most altered (especially when conditioning with melphalan)	- CG (2 concentrations)
Larsen et al. 2004 [25]	Different	Allo + Auto	43	- T0 (week before admission): 17% - T1 (day before conditioning regimen): 31% - T2 (day of SCT): 55% - T3 (start of protective care period): 58% - T4 (mid-point of protective care period): 54% - T5(end of protective care period): 53% - T6 (day of discharge): 42%	Adults	- Change of taste was reported by more than 50% of the patients during T2-T5	- Self-report (SFID-SCT)
Majorana et al. 2015 [26]	Different	Not specified	51	- BL (hospital admission): - - Twice during conditioning therapy: - - 3 and 6 months: -	Children (3-12y)	- Changes in taste perception seem to occur especially during the conditioning therapy and resolve in about six months post HCT	- CG
Patel et al. 2023 [27]	Different	Allo	66	BL (pre-HCT): 20% Post HCT (worst PRO between day 14 to day 100): - Grade 0–1: 66% - Grade 2–4: 69%	Adults	- Taste loss is most frequently reported symptom at any time point within the first 100 days (88%) (with fatigue) - “severe” and “very severe” lack of taste affected 31% of acute GVHD patients and 19% for no GVHD	- Self-report (PRO-CTCAE)
Scordo et al. 2022 [28]	HDM	Auto	45	- BL (pre-HCT): 29 - Day -1: 30 - Day 7: 27 - Day 14: 29 - Day 30: 28 - Day 100: 29 (CG median scores) **	Adults	- Lowest scores (highest dysgeusia) for each taste was between day 7 and 14, corresponding to NP - Recovery of dysgeusia occurred between day 30 and 100 - Negative correlations between lower CG scores and higher symptom burden (STTA and PRO-CTCAE)	- CG - Self-report (STTA and PRO-CTCAE)
Uyl-de Groot et al. 2005 [29]	Before first HCT: - Two courses of VAD or VAMP - HDM Before second HCT: - Busulfan/cyclophosphamide	Double HCT	51	- BL (pre-HCT): 20 - T2 (hospital discharge after HDM + first HCT): 23 - T3 (1 month after discharge): 6 - T4 (day of hospital admission): -9 - T5 (day of discharge after HCT): 21 - T6 (6 months after hospital discharge): -4 - T7 (12 months after discharge): -8 (self-report) ***	Adults	- Considerable increase in patient-reported change of taste shortly after HDM and PSCT - None of the symptoms persisted during follow-up	- Self-report (customized questionnaire)
Wysocka-Slowik et al. 2021 [30]	Different (divided in MAC or RIC)	Allo	80	- BL (pre-HCT): 11% - Day 3–7: 20% - Day 8–14: 20%	Adults	- Dysgeusia was the fourth most commonly reported subjective oral complaint	- Self-report (customized questionnaire)

Abbrevations: *BL* baseline, *CG* chemical gustometry, *MAC* myeloablative conditioning, *RIC* reduced intensity conditioning, *TBI* total body irradiation, *HDM* high dose melphalan, *VAD* vincristine adriamycin dexamethasone, *VAMP* vincristine adriamycin methyl prednisone, *HCT* hematopoietic cell transplantation, *NP* neutropenic phase

^*^ Range 0–100, higher scores indicate higher level of symptoms

^**^ Range 0–30, higher scores indicate better recognition of taste

^***^ Mean absolute score at BL and mean change scores from BL, a negative change score reflects a lower level of symptoms

Eight studies included different types of conditioning regimens [20, 21, 23–27, 30], whereas three studies [22, 28, 29] only included one conditioning protocol, described in detail in Table 1. Among studies including different types of conditioning regimens, two studies divided conditioning into a myeloablative conditioning (MAC) and reduced-intensity conditioning (RIC) group [21, 30]. Types of transplants included only allogeneic in three studies [21, 27, 30], only autologous in one study [28], and both allogeneic and autologous in four studies [20, 23–25]. One study included double transplantation [29] and 2 studies did not report the type of transplant used [22, 26].

Taste was assessed objectively, with chemical gustometry, in six studies [20, 22–24, 26, 28]. Subjective taste was assessed in eight studies, with various questionnaires [20, 21, 25, 27–30] or verbally [22]. The types of questionnaires used are described in Table 1.

### Prevalence of dysgeusia

#### Pre-engraftment period

Baseline measurements of taste included assessments before HCT, either before the start (i.e. hospital admission) or during conditioning therapy. Only one study assessed taste in adults prior to hospital admission, with a prevalence of 17% of self-reported dysgeusia [25].

Self-reported baseline (i.e. before HCT) dysgeusia ranged between 11 and 31% in adults [25, 27, 30] and was 0% in children [22]. According to objective measurements, dysgeusia was prevalent in 2.4% of adults [24] and 10% of children [23]. There was no difference in taste score between the RIC and MAC group at baseline [21]. Before HCT, there were few self-reported [20, 29] and objective [28] taste alterations in adults. According to one study in children, objective changes in taste occurred during conditioning therapy, with increased threshold values for all four flavors [26].

Dysgeusia during the neutropenic phase (i.e. 7 to 14 days after the start of conditioning) until discharge, was reported in five studies [22, 24, 25, 28, 30]. Self-reported prevalence of dysgeusia during the neutropenic phase ranged between 20 and 58% in adults [25, 30] and was 100% in children [22]. According to objective measures, dysgeusia prevalence was 21.4% in adults [24]. Subjective and objective changes in taste in adults were most prevalent during this period [24, 25, 28, 30].

#### Early and late post-engraftment period

Six studies reported taste shortly after neutrophil recovery until approximately one month after HCT (± 30d) [20–23, 28, 29], six studies more than 1 month later (± 100d) [20, 23, 26–29] and 2 studies until a year later [21, 29].

Reported dysgeusia prevalence in children, approximately one month after HCT, was 33% according to objective measures [23], compared to 18% based on self-report [22]. In adults, at day 30, self-reported symptoms were still more elevated than baseline [20, 21, 29]. However, in one study there was a decreasing trend in self-reported symptoms [29]. At one month after HCT, there were more self-reported taste alterations in the MAC group compared to the RIC group [21].

Taste sensitivity seems largely recovered in adults by day 80 according to self-report [20] and day 100 according to objective measures [28]. At six and 12 months after receiving a second HCT, self-reported complaints of dysgeusia in adults were less than at baseline [29]. According to objective measures of taste, dysgeusia prevalence in children is 0% at 2 months after HCT [23]. Another study in children reports recovery of objective taste between 3 and 6 months after HCT [26]. The prevalence of self-reported dysgeusia was slightly higher in grade 2–4 GvHD (69%) compared to grade 0–1 GvHD patients (66%) during follow-up [27]. Self-reported dysgeusia decreased in both RIC and MAC groups between 1 and 12 months after HCT [21].

### Characteristics of dysgeusia

#### Sweet

Sweet taste, before and after HCT, was objectively measured in six studies [20, 22–24, 26, 28]. At baseline, upon admission, there was no difference in sweet threshold between patients and controls [22]. During conditioning therapy and the neutropenic phase, there was a decrease in sensitivity for sweet [24, 26]. After engraftment, there was no difference in sweet taste compared to baseline [22, 23, 26, 28]. In one study there was even an increased taste sensitivity for sweet at day 30 and day 80 [20]. Only 13% of patients had an abnormal sweet taste on day 100 compared to 16% at baseline [28].

#### Sour

Sour taste, before and after HCT, was objectively measured in six studies [20, 22–24, 26, 28] At baseline, upon admission, there was a significant difference in sour threshold between patients and controls [22]. During conditioning therapy and the neutropenic phase, there was a decrease in sensitivity for sour [24, 26]. After engraftment, no difference in sour threshold was observed compared to baseline [22, 23, 26, 28]. Furthermore, 10% of patients had an abnormal sour taste on day 100, compared to 14% at baseline [28].

#### Bitter

Bitter taste, before and after HCT, was objectively measured in six studies [20, 22–24, 26, 28]. At baseline, upon admission, there was no significant difference between patients and controls [22]. Before conditioning, throughout the neutropenic phase and after engraftment, there was no difference in bitter thresholds compared to BL [20, 22–24, 28]. In fact, bitter scores were the least frequently reduced of all four tastes, with only 3% of patients with abnormal bitter taste at day 100, compared to 14% at baseline [28]. However, in one study [26] there was a decreased sensitivity for bitter during conditioning therapy, but this returned to baseline values after engraftment.

#### Salt

Salt taste, before and after HCT, was objectively measured in six studies [20, 22–24, 26, 28]. There was no significant difference between patients and controls at admission [22]. There was a decreased taste sensitivity for salt during conditioning and the neutropenic phase [22, 24, 26]. After engraftment, there was no significant difference in taste thresholds compared to baseline values [22, 23, 28]. In one study, taste sensitivity for salt only returned to baseline values on day 80 [20]. However, 32% of patients had an abnormal salt taste on day 100, compared to 14% at baseline [28].

#### Umami

Umami taste, before and after HCT, was only reported in one study [28]. In this study, overall umami sensitivity remained the same between baseline and 100 days after HCT. However, umami taste scores were most frequently reduced, with up to 49% of patients having an abnormal umami taste at day seven until day 30. Additionally, up to 36% of patients had an abnormal umami taste that persisted on day 100 compared to 43% at baseline.

## Discussion

Patients undergoing HCT may develop dysgeusia as a complication of treatment. However, it is not clear to what extent these taste changes are prevalent and the conditions in which they occur. This scoping review sought to map current knowledge in the literature including the prevalence and characteristics of dysgeusia after HCT. We found that dysgeusia may already be present before HCT, and that highest complaints occur during the neutropenic phase. While taste alterations seem to be still elevated in the early post-engraftment period, they appear to largely recover in the late post-engraftment period. Furthermore, all basic tastes, except for bitter, seem to be altered during treatment. Most affected tastes are umami and salt. Amongst taste change, umami may impact oral intake, dietary choices, and enjoyment of taste and appetite.

In this review we found that some participants already had taste alterations prior to treatment. Several factors could account for these pre-existing taste alterations, including earlier cancer treatment, direct influence of the disease and the use of supportive medications [6, 8]. Furthermore, antibiotics or drugs preventing GvHD (e.g. cyclosporine or mTOR inhibitors) may have a negative effect upon taste [31]. This raises the question to what extent the taste disorders experienced during HCT can be attributed specifically to the effect (of the conditioning) of HCT.

Taste alterations, objective as well as subjective, seem to be worst during the neutropenic phase, implying direct impact of the conditioning regimen. In fact, conditioning-related taste alterations are typically related to the onset of oral mucositis and suggest a direct interference between the toxic drugs of the conditioning regimen and taste receptor cells [13, 30]. Other possible (indirect) factors include oropharyngeal mucosal infections, neurologic toxicity (affecting the taste and smell pathway) and saliva characteristics [30]. Higher melphalan concentrations in saliva correlated with worse patient-reported dysgeusia suggesting local toxicity [28]. As most (oral) complications after HCT develop in clusters [13], it remains difficult to identify the exact relations and mechanisms.

On the other hand, taste seems to recover after HCT, when the conditioning regimen has stopped (i.e. chemotherapy), indicating conditioning-related toxicity. Taste cells rapidly renew when treatment is finished, resulting in recovery of taste [26]. In cases where taste does not rapidly come back and new or recurrent taste changes occur, a possible reason may be the onset of chronic oral GvHD in allogeneic HCT recipients [13, 32]**.** Most symptoms, such as tiredness, mouth dryness, loss of appetite as well as dysgeusia, recover during follow-up [21, 25]. This further confirms the hypothesis of dysgeusia being a part of a broader set of interrelated adverse events that develop in clusters [13, 28].

Only one study evaluated umami taste. Umami seemed to be most affected even though limited research about this taste has been performed. A possible reason is that umami has only recently been recognized as one of the basic tastes. However, umami is important for palatability and enjoyment of food and therefore may play a crucial role in QoL and appetite [33, 34]. Furthermore, umami also seems to be most affected on the long-term in allogeneic transplant recipients [32].

Across the selected studies, there was high heterogeneity in taste assessment. Different methods for the assessment of taste were used, including objective and subjective measures. Both methods are valuable as objective measures are useful to understand the physiology of taste alterations, while subjective measures may be more reliable to predict changes in diet and QoL as they reflect a patient’s experience [15]. Furthermore, there is discrepancy between subjective and objective prevalence of dysgeusia. Indeed, some patients having objective dysgeusia, may be unaware of their dysfunction, therefore not reporting dysgeusia and vice versa. Self-perception of chemosensation is driven by many factors (e.g. age, persistent cold symptoms) which may lead to inaccurate estimation of the actual dysfunction [35].

Not only were there differences between objective and subjective measures, but variations also existed within each of these measurement methods. For instance, within subjective measures, multiple validated questionnaires (e.g. European Organization for Research and Treament for Cancer Core Questionnaire (EORTC QLQ-C30) or High-dose chemotherapy questionnaire (HDC-19)) with different questions and scales were used. Moreover, within objective measures, differences in assessment strategy (e.g. number of concentrations) were seen. These variations may account for the differences in prevalence of dysgeusia across studies and ultimately lead to difficulties interpreting the severity of dysgeusia after HCT. Other contributing factors for these variations may be differences in sample size or sample characteristics (i.e. previous treatments, conditioning, age, gender).

In this review it seems that children have less subjective and objective dysgeusia at baseline and that objective recovery of taste is faster in children compared to adults. In fact, taste alterations are less common in children, and they may recover more rapidly after treatment [23, 36]. Unfortunately, due to differences in follow-up, sample sizes and assessment strategies, it is difficult to directly compare results in adults and children, therefore making it impossible to draw definitive conclusions.

Baseline data of taste was valuable, as it made comparison of taste prior and after treatment possible. This gave a unique insight into the extent of injury and recovery during and after treatment. However, there was heterogeneity in the time-points of taste assessment. Baseline taste measurements included taste before the start of the conditioning regimen in some studies whereas in other studies it was measured when the conditioning had already started. Furthermore, frequency and timing of taste assessment at follow-up varied. This made it difficult to compare trends and prevalence across studies and map the course of dysgeusia complaints.

Future studies should explore the effect of taste alterations on nutrition, as specific taste disturbances may lead to specific food aversions, nutritional compromise, and delayed recovery. Systemic factors, such as the type of conditioning or the use of immunosuppressants, as well as local factors, such as hyposalivation, GvHD and oral mucositis are common findings in the early phases of HCT treatment, potentially influencing dysgeusia following HCT. These factors should be assessed in future studies to clarify their impact. Dysgeusia after HCT may be studied in homogenous patient populations with the combination of objective and subjective taste measures in order to identify influencing factors and mechanisms of dysgeusia. Further unraveling of the prevalence, nature and mechanisms underlying dysgeusia will ultimately lead to improved, more targeted interventions.

## Conclusion

Some patients undergoing HCT experience dysgeusia with highest complaints occurring during the neutropenic phase. However, taste seems to largely recover in the post-engraftment period. All basic tastes, except bitter, seem to be affected. Umami and salt were most affected by treatment. The lack of standardized assessment methods prevents generalizability of the results.

## Supplementary Information

Below is the link to the electronic supplementary material.

Supplementary file1 (DOCX 34 KB)

## Data Availability

No datasets were generated or analysed during the current study.
