# Supplementary material for: Taste alterations after hematopoietic cell transplantation: a scoping review
Source: Support Care Cancer. 2024 Sep 25;32(10):687. doi: 10.1007/s00520-024-08900-w (PMC11424654; doi:10.1007/s00520-024-08900-w)
Supplement: Supplementary file 1 — Supplementary file1 (DOCX 34 KB) [file 520_2024_8900_MOESM1_ESM.docx]

**PubMed Session Results (22 Jun 2023)**

| Search | Query | Items found |
| --- | --- | --- |
| #3 | **#1 AND #2** | 263 |
| #2 | **"Taste"[Mesh] OR "Taste Perception"[Mesh] OR "Taste Buds"[Mesh] OR "Taste Disorders"[Mesh] OR "dysgeusi*"[tiab] OR "ageusi*"[tiab] OR "parageusi*"[tiab] OR "hypogeusi*"[tiab] OR "hypergeusi*"[tiab] OR "metallogeusi*"[tiab] OR "cacogeusi*"[tiab] OR "phantogeusi*"[tiab] OR "aftertast*"[tiab] OR "taste"[tiab] OR "tastes"[tiab] OR "tasting"[tiab] OR "gustat*"[tiab]** | 57,083 |
| #1 | **"Stem Cell Transplantation"[Mesh] OR "stem cell*"[tiab] OR "cell transplant*"[tiab] OR "HSC"[tiab] OR "HSCT*"[tiab] OR "alloHSC"[tiab] OR "alloHSCT*"[tiab] OR "SCT"[tiab] OR "alloSCT*"[tiab] OR "Bone Marrow Transplantation"[Mesh] OR (("bone marrow"[tiab] OR "hematopoetic"[tiab] OR "haematopoetic"[tiab] OR "hematopoietic"[tiab] OR "haematopoietic"[tiab] OR "hemopoietic"[tiab] OR "haemopoietic"[tiab] OR "hemopoetic"[tiab] OR "haemopoetic"[tiab]) AND ("transplant*"[tiab] OR "graft*"[tiab] OR "transfus*"[tiab] OR "transfer*"[tiab]))** | 444,447 |

**Embase.com Session Results (22 Jun 2023)**

| Search | Query | Items found |
| --- | --- | --- |
| #4 | **#3 NOT ('conference abstract'/it OR 'conference review'/it)** | 625 |
| #3 | **#1 AND #2** | 860 |
| #2 | **'taste'/exp OR 'taste bud'/exp OR 'taste disorder'/exp OR 'dysgeusi*':ab,ti,kw OR 'ageusi*':ab,ti,kw OR 'parageusi*':ab,ti,kw OR 'hypogeusi*':ab,ti,kw OR 'hypergeusi*':ab,ti,kw OR 'metallogeusi*':ab,ti,kw OR 'cacogeusi*':ab,ti,kw OR 'phantogeusi*':ab,ti,kw OR 'aftertast*':ab,ti,kw OR 'taste':ab,ti,kw OR 'tastes':ab,ti,kw OR 'tasting':ab,ti,kw OR 'gustat*':ab,ti,kw** | 86,112 |
| #1 | **'stem cell transplantation'/exp OR 'stem cell*':ab,ti,kw OR 'cell transplant*':ab,ti,kw OR 'HSC':ab,ti,kw OR 'HSCT*':ab,ti,kw OR 'alloHSC':ab,ti,kw OR 'alloHSCT*':ab,ti,kw OR 'SCT':ab,ti,kw OR 'alloSCT*':ab,ti,kw OR 'bone marrow transplantation'/exp OR (('bone marrow':ab,ti,kw OR 'hematopoetic':ab,ti,kw OR 'haematopoetic':ab,ti,kw OR 'hematopoietic':ab,ti,kw OR 'haematopoietic':ab,ti,kw OR 'hemopoietic':ab,ti,kw OR 'haemopoietic':ab,ti,kw OR 'hemopoetic':ab,ti,kw OR 'haemopoetic':ab,ti,kw) AND ('transplant*':ab,ti,kw OR 'graft*':ab,ti,kw OR 'transfus*':ab,ti,kw OR 'transfer*':ab,ti,kw))** | 681,309 |

**Web of Science (Core Collection) Session Results (22 Jun 2023)**

| Search | Query | Items found |
| --- | --- | --- |
| #3 | **#1 AND #2** | 378 |
| #2 | **TS=("dysgeusi*" OR "ageusi*" OR "parageusi*" OR "hypogeusi*" OR "hypergeusi*" OR "metallogeusi*" OR "cacogeusi*" OR "phantogeusi*" OR "aftertast*" OR "taste" OR "tastes" OR "tasting" OR "gustat*")** | 89,039 |
| #1 | **TS=("stem cell*" OR "cell transplant*" OR "HSC" OR "HSCT*" OR "alloHSC" OR "alloHSCT*" OR "SCT" OR "alloSCT*" OR (("bone marrow" OR "hematopoetic" OR "haematopoetic" OR "hematopoietic" OR "haematopoietic" OR "hemopoietic" OR "haemopoietic" OR "hemopoetic" OR "haemopoetic") AND ("transplant*" OR "graft*" OR "transfus*" OR "transfer*")))** | 633,385 |
